# Evaluation of nano-confined catalytic oxidation air purification technology on eliminating marijuana chemicals and odour

**DOI:** 10.1007/s42452-021-04783-2

**Published:** 2021-09-27

**Authors:** Abiel Kwok, Christopher Hong, Ezra Kwok

**Affiliations:** Department of Chemical and Biological Engineering, University of British Columbia, Vancouver, BC Canada

**Keywords:** Indoor air quality, Air pollution, Cannabis, Catalytic oxidation, Zeolites

## Abstract

The novel air purification technology, Nano-Confined Catalytic Oxidation (NCCO), has been proven to be effective at eliminating air pollutants. With the increasing legalization and decriminalization of medicinal and recreational cannabis and related products, respectively, in many countries and jurisdictions around the world, concerns have been raised about indoor air quality from smoking cannabis products, such as marijuana, which produce gaseous pollutants and intense odour. In this study, NCCO technology has been evaluated for its effectiveness in reducing key marijuana concentrations in polluted indoor air by direct measurements and odour intensity assessments by human volunteers. For the odour intensity measurements, 20 non-cannabis adults participated in the odour assessment. The results are remarkable and statistically significant. The reduction in Dronabinol, a pharmaceutical form of Tetrahydrocannabinol (THC), Cannabidiol (CBD) and Cannabinol, averages 93.4%, whereas that in airborne marijuana compounds with no air purification only averages 6.2%. The technology also demonstrates statistically significant reductions in PM2.5, PM10 and total volatile organic compounds generated from marijuana smoke. The technology was able to restore high levels of harmful particulate matter to normal baseline levels. Furthermore, the odour assessment conducted by a group of 20 volunteers also confirmed statistically significant reductions in marijuana odour by 55.6% after 50 min of air purification.

## Introduction

Cannabis and related products are becoming more widely used worldwide. The United Nations Office on Drugs and Crime (UNODC) estimates 192 million people consumed cannabis globally in the past year and the number has been continuously on the rise for more than two decades [1, 2]. The legality of cannabis for medical and recreational use varies by country and jurisdiction. UNODC reported a total of 33 States and four Districts had legalized medical cannabis by the end of 2019. Around the world, over 35 countries have legalized cannabis and/or related products for medicinal use and decriminalized recreational use. With the legalization of marijuana in Canada in 2018 and more countries and jurisdictions following the trend, growing concerns have been raised on the impact of marijuana use on indoor air quality (IAQ). More than 400 chemical species have been identified in the cannabis plant (*Cannabis sativa L.*). Among them, the two major classes of compounds Tetrahydrocannabinol (THC) and Cannabinoid (CBD) have been of primary health concern [3]. The secondary concern is raised on the intense odour produced by cannabis combustion [4].

The current regulations on IAQ concerning cannabis are limited to the context of indoor and outdoor cannabis production. Health Canada and other provincial authorities have focused on eliminating odour through the removal of volatile organic compounds (VOC) in plantation facilities [5, 6]. The use of H13 high-efficiency particulate air (HEPA) filter and maintenance of sufficient ventilation are recommended by Health Canada’s regulatory documents [7]. The impact on health and quality of living for the non-users exposed to the chemicals and odour have not been properly addressed. This will continue to be a significant issue, especially in the high-density urban living area such as apartment building and townhomes.

The passive exposure to cannabis has been investigated extensively by the medical community. Both THC and CBD have been demonstrated to accumulate in hairs and blood after exposure to second-hand cannabis smoke [8, 9]. Additionally, due to the nature of many VOCs adsorbing to exposed surfaces and subsequent release back into the air in confined spaces, the third-hand exposure without being in proximity with an active smoker has also raised many concerns on the effectiveness of air treatment on cannabis smoke [10].

The IAQ assessment and treatment in the context of cannabis smoking have rarely been investigated. A number of air purifiers using different technologies such as activated carbon, ionization, ultraviolet light, or even chemical oxidizer are available to consumers. All these products claim to be effective for air purification. However, common indoor air pollutants include a wide variety of particulate matter (PM) and gaseous contaminants. The next section provides an overview of these widely known technologies which cannot handle all common indoor air pollutants. Furthermore, no formal testing or evaluation of these technologies has been conducted for cannabis smoke and chemical removal. Recently, Nano-Confined Catalytic Oxidation (NCCO) technology has demonstrated high effectiveness in improving IAQ in all categories of air pollutants. In 2012, Leung and Kwok demonstrated that NCCO outperformed two other commonly used air purifiers in reducing odour nuisances caused by ammonia, toluene and hydrogen sulphide [11].

The objective of this project is to evaluate the effectiveness of an NCCO air purifier to reduce cannabis odour, smoke, and chemicals represented by the air concentration of THC and CBD.

In this paper, the methods of evaluation will be explained in the following section. This is not a comparative study because it is difficult to compare air purifiers with different technologies. The efficacy of different technologies is dependent on different factors such as energy usage, exposure time, or active sites. In order to evaluate the efficacy of this NCCO technology objectively, the study will examine the main chemical concentrations representing marijuana, standard IAQ parameters and also odour assessment from volunteers. The results are presented in graphical forms, followed by a discussion of the results.

## Air purification technologies

### Common technologies

The common methods for air cleaning include mechanical filtration, ultraviolet germicidal irradiation (UVGI), electrostatic precipitation, electrostatic ionization, sorption, ozone generation, and photocatalytic oxidation (PCO) [16]. Some technologies also are available for large-scale industrial applications for deodorization of malodorous gases [17, 18]. Most residential air cleaners use a combination of purification strategies to enhance performance. Leung and Kwok (2012) provided an overview of these technologies. The following explains their key functions and limitations for IAQ improvement:

- **Mechanical filtration**
  A High-Efficiency Particulate Air (HEPA) filter is a type of mechanical air filter which can remove suspended particulates. Various types of filters can be rated on the minimum efficiency reporting value (MERV) scale of trapping more than 99.97% of airborne particles at 0.3 μm. However, it cannot remove gaseous pollutants such as formaldehyde and total volatile organic compounds (TVOCs). It also does not have any disinfection property. In heavily polluted environment, the lifetime of the HEPA filter will be shortened dramatically.
- **Ozone**
  High concentration of ozone (O_3_) is very effective in oxidizing indoor pollutants for disinfection and deodorization. However, Excessive ozone exposure and high ozone concentration lead to a few health issues. When ozone concentration is maintained within public health standards, the oxidation reaction with most common pollutants is relatively slow. The end result is ineffective decomposition of gaseous phase pollutants.
- **Electrostatic precipitation/ionization**
  This technology removes pollutants by air ionization. There are various ways to generate ions; a typical method involves applying a voltage between a sharp electrode and a plate. Secondary pollutants such as ozone and NO_x_ are formed during the electrical discharge. They constitute a major disadvantage of ionizing air cleaners. In the precipitation method, airborne particulates are first charged by attraction towards oppositely charged collection plates. To maintain optimal performance of these units, the collection plates must be cleaned periodically to remove build-up. In contrast, ionization generally does not contain collection plates. Instead, ions are emitted into the environment to charge airborne particles, which adhere to nearby surfaces (e.g. walls, ceiling, and furniture) by electrostatic attraction. Moreover, the ion will decay very quickly. This technology is not effective for applications in large areas.
- **Activated charcoal**
  In general, activated carbon filters are known to be very effective and should be able to absorb chemical and odour in the air. However, there are a few significant limitations. First, the filter performance is greatly reduced by increased humidity. Second, activated carbon is ineffective against particular matters, bacteria, viruses, and nonhydrocarbon compounds. Third, activated charcoal will have a problem of saturation and its efficiency will quickly diminish and become even useless once saturated. Fourth, there will also have a chance of secondary pollution when the adsorbed pollutants are re-released back into the environment after saturation. Fifth, maintaining high efficiency requires frequent filter replacement. The disposal of used activated carbon filters becomes perpetuates the pollution of our environment.
- **UV light**
  UV light achieves its sterilizing function by causing nucleic acid damage and inhibits replication of organisms such as bacteria and viruses. However, the penetration power of residential UV light is very low. Some pathogens also exhibit reduced susceptibility to UV due to the presence of protective coverings, such as cell walls. When the organisms are blocked by dust or other material, UV light cannot show any performance at all. The ability for UV light to decompose gaseous phase pollutants is also very limited.
- **Photo-catalytic oxidation (PCO)**
  PCO is a combination of a photo source with a catalyst (normally titanium oxide). The wavelength for the UV light has to be in the suitable range (normally UV light). There is limited ability for decomposing gaseous phase pollutants and disinfection. The speed/rate of reaction is also slow. The treatment usually takes a long time. When the surface of the catalyst is polluted, no light can be absorbed. The device quickly loses its performance.

### Nano-confined catalytic oxidation technology

NCCO air purification technology has been patented and utilizes the oxidizing strength of active oxygen and zeolite’s adsorptive and catalytic properties to absorb and oxidize air pollutants [19]. Active oxygen is a generic term for air molecules which are altered from atmospheric oxygen into a chemical compound with more reactive characteristics. Active oxygen possesses very strong oxidizing properties effective at neutralizing pollutants. However, active oxygen also contains ozone. The high emission of ozone to the surrounding environment and complicated ozone chemistries leading to undesired reaction intermediates have discouraged the use of high-concentration ozone alone in air purification [12]. Zeolites are aluminosilicate minerals with nano-sized pores and serve as a strong absorbent to ozone and other VOCs and pollutant. By coupling low-concentration of active oxygen and zeolites coated with selected transition metals, pollutant and low-concentration of active oxygen are confined into pores optimal for the catalytic oxidation of pollutants to H_2_O and CO_2_. Additionally, this combination retains reaction intermediates and excess ozone within the zeolite channels, preventing undesired ozone or chemical emission into neighbouring air [13]. The following block diagram illustrates the design of a NCCO air purifier:
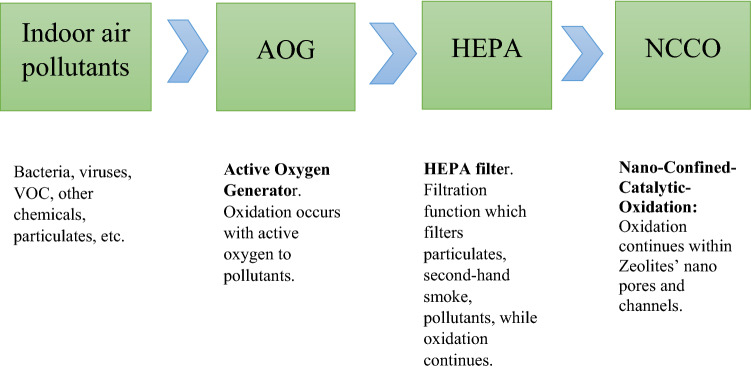


The overall NCCO technology is to directly decompose air pollutants into mostly water and carbon dioxide using active oxygen with a very low ozone concentration which is within public health standards. The following is a list of advantages:

- Energy saving. There is no chemical extraction, air dilution or exhaust.
- Cost efficient. The NCCO reactor does not have any saturation problem, different from activated carbon.
- No secondary pollution or re-release of pollutants.
- Effective against indoor air pollutants including airborne organisms, volatile organic compounds, other chemicals, fine particulate matters, ozone, carbon monoxide, nitrogen dioxide, etc.
- Environmentally friendly by having less solid waste. The Zeolite particles can be used theoretically indefinitely. Even when the zeolite reactor needs to be replaced due to natural deposits over years, the zeolite particles are not harmful to the environment. Unlike zeolites, activated carbon filters still carry accumulated air pollutants.

The only disadvantage of the NCCO technology is its high static pressure drop across the NCCO reactor.

## Methods

This study aims to evaluate the effectiveness of NCCO air purification technology on eliminating odour and chemical pollutant produced by cannabis smoke. The study includes two major components: chemical component analysis and a single-blind relative odour intensity evaluation experiment on indoor air with or without NCCO purification.

### NCCO air purifier

Several brands of NCCO-based air purifiers are available for commercial and residential use. The current study employed a residential NCCO air purifier that is suitable for a room up to 500 square feet. The machine has a dimensions of 510 (L) x 260 (W) x 893 (H) mm and an air flow volume of 180 m^3^/h (lowest), 700 m^3^/h (highest) and 780 m^3^/h (turbo).

### Cannabis

THC-CBD *Indica* cannabis (total THC 4.975% w/w, total CBD 9.610% w/w) is weighed into 1 g portions, grounded and rolled into individual joints. Each joint is stored individually in glass jars with moisturizing packs supplied by the dispensary and stored away from light and heat.

### Chemical concentration and air quality assessment

One of the major goals of the study is to evaluate the effectiveness of the elimination of THC and CBD, the two pharmacologically significant components of cannabis, by NCCO technology. The concentration of Dronabinol, a pharmaceutical form of THC, and two types of CBD, Cannabidiol and Cannabinol, are assessed before and after 30 minutes of air purification or 10 room air turnovers at the highest setting (700 m^3^/h).

Air samples are collected in a 3 m x 4.5 m x 4.5 m room covered with HDX plastic sheeting. At each sampling time, three air samples are collected from three locations inside the room. The air samplers are partially bordered by cardboards which reduce air turbulence during air sample collection. The air sampling uses Casella Apex air sampling pumps calibrated for each sorbent collection tube to 2 L/min using Bios defender 510 air flow calibrator.

Before the start of the experiment, one baseline room air sample is collected into a sorbent tube to confirm the absence of air marijuana pollutants. Then, a joint is attached to an air pump before being lighted. The air pump facilitates the generation of marijuana smoke which fills the entire room. Three air samples are then collected immediately for 10 minutes at 2 L/min to establish the initial concentrations. The NCCO air purifier is then turned on. After 20 minutes of air purification, three more air samples are collected for 10 minutes at 2 L/min while the air purifier is on. The second sampling determines how much the chemical concentrations drop. After the second set of samples is collected, the NCCO air purifier continues to run for another 30 min for a total of one hour. During the experimental period, IAQ parameters including PM2.5, PM10, CO_2_, total VOC (TVOC), temperature and humidity are collected by Air Mentor 2 Indoor Air Quality Detector (8099-AP). The device has the following detection range limits:

- PM2.5 and PM10: 0–1000 µg/m^3^
- TVOC: 0.020–10 ppm
- CO_2_: 400–10,000 ppm
- Temperature: − 20 °C– 80 °C
- Humidity: 0–100%

Its accuracy and consistence performance can be found at the manufacturer’s website [20].

The whole procedure above is repeated without any air treatment as a control experiment. Between the first experiment and the control experiment, the room is completely aired out and restored to baseline which is confirmed by the Air Mentor 2 monitor.

The concentration of Dronabinol, Cannabidiol and Cannabinol is quantified using gas chromatography and mass spectrometry as follows:

- Standardize the Analytical Reagent (AR) grade DCC solutions by using Certified Reference Material (CRM).
- Prepare the calibration curve of Dronabinol, Cannabidiol and Cannabinol by using AR grade Dronabinol, Cannabidiol and Cannabinol solutions.
- Collect the air quality samples by using sorbent collection tubes.
- Use a Gas chromatography – Mass spectrometry (GC–MS) machine for identifying the concentration of Dronabinol, Cannabidiol and Cannabinol.

For identifying Dronabinol, Cannabidiol and Cannabinol, a size of 25 m x 0.2 mm, 0.33μm film thickness of fused-silica capillary column was used for chromatographic separation. Ultra-high purity of Helium was used as the carrier gas with 1 mL/min flow rate. Certified Reference Materials (CRM) of Dronabinol, Cannabidiol and Cannabinol were used as a standard for comparative analysis with the air quality samples.

### Relative odour intensity assessment

To evaluate the efficiency of eliminating odour with NCCO technology, a relative scale of 0–5 shown in Table 1 is designed to assess the intensity of cannabis odour in the room. Due to the fact many components of cannabis are prone to adhere to the walls, the score 1 is set to be the baseline cannabis smell of the room after 30 minutes of ventilation, while 0 represents the room air with no smell and 5 representing the saturated odour immediately after a joint is burnt.

**Table 1:** Relative odour intensity scale

0	No smell
1	Baseline smell
2	Weak smell
3	Medium smell
4	Strong smell
5	Saturated smell

Twenty adults volunteered to particulate in the single-blind odour test. All volunteers were informed of the set-up of the experiment. They also provided informed consent to the study. None of the volunteers has previous experience with cannabis use. The single-blind test is performed in the same 3 m x 4.5 m x 4.5 m room covered with HDX plastic sheeting, with an additional partition at the entrance with the plastic sheet to minimize disturbance to indoor air by volunteer opening and closing the room door. Prior to the start of the test, a joint is burnt and puffed with an air pump in the room followed by 30 minutes of high throughput ventilation; this condition is defined as the baseline smell. The reason is that odour chemicals tend to attach to surfaces and generate odour even though the room has been aired. A ventilated room with previous marijuana use is defined as the baseline odour smell. The volunteers were all exposed to the maximum odour level (defined as 5) and baseline odour level (defined as 1) before starting the experiment and control. Another joint is burnt and puffed completely, with this condition being defined as the saturated smell or maximum odour level. Volunteers were asked to enter the room, walk clockwise around the room and smell-assess the odour intensity based on the given baseline and saturated level after 25 and 50 minutes of treatment by the NCCO unit. During the assessment, the NCCO unit is turned off to ensure the single-blind design of the experiment.

The above procedure is then repeated without NCCO treatment. The relative intensity is then analysed using one-way ANOVA test.

The volunteers were not informed of when the NCCO air purifier was used to treat the air. This arrangement made this experiment a single-blind study.

## Results

All data generated or analysed during this study are included in this published article.

### Chemical concentration analysis

**Fig. 1:**
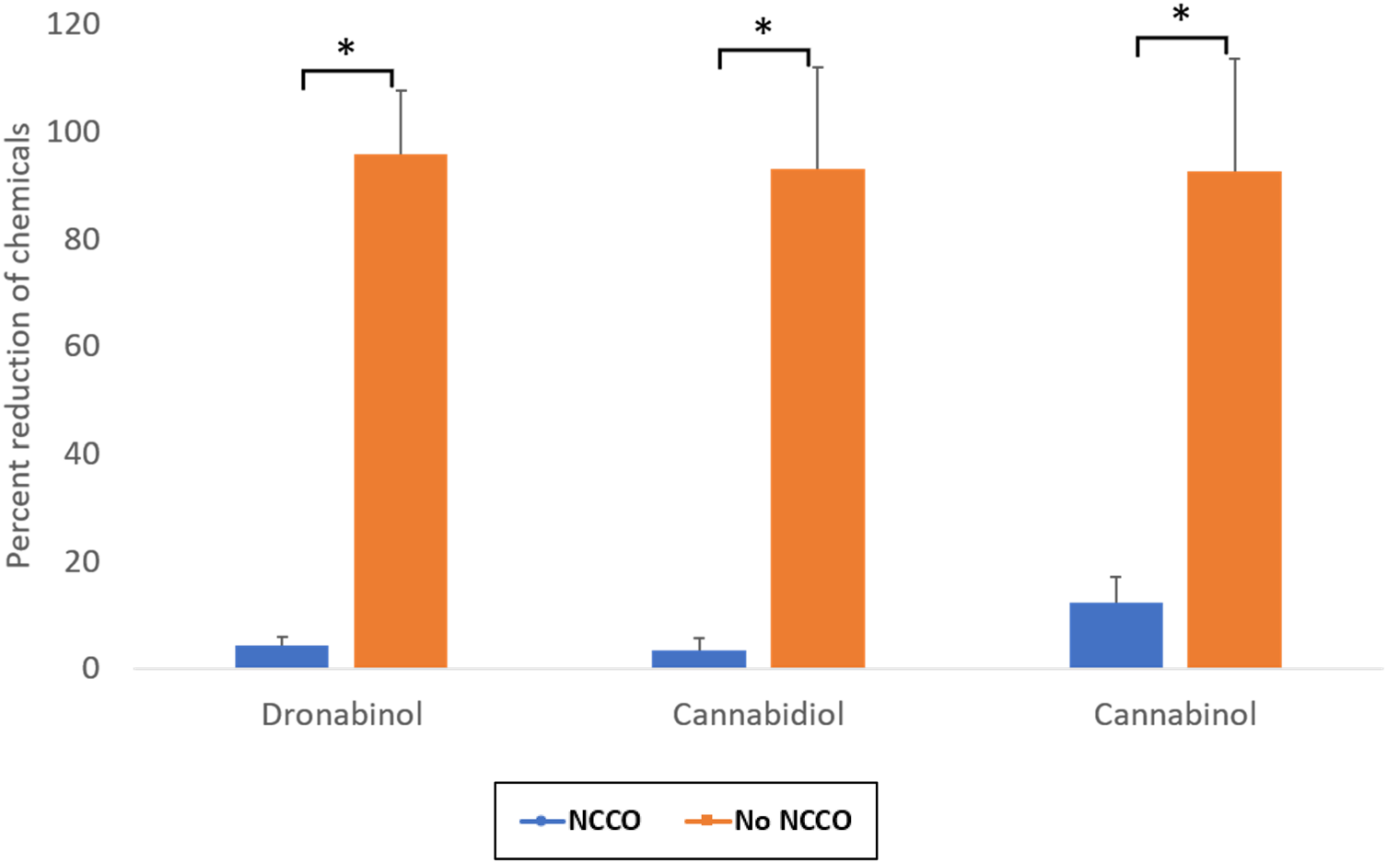
Summary of percentage reduction for Dronabinol, Cannabidiol and Cannabinol after 30 min of NCCO treatment. Error bar represents 1 SEM. *p < 0.05

The three compounds produced by cannabis, Dronabinol, Cannabinol and Cannabidiol, are quantified using GC-MS. The change in absolute area and percentage decreases in chemical concentration are presented in Table 2 and Fig. 1, respectively.

**Table 2:** Air concentration of marijuana chemicals and percentage reduction after 30 min

	GC/MS area response			% reduction		
Description	Dronabinol	Cannabidiol	Cannabinol	Dronabinol	Cannabidiol	Cannabinol
Baseline Sample	0	0	0			
0 min Sample A	177,251	2,911,571	1,155,257			
0 min Sample B	196,908	2,936,951	1,405,781			
0 min Sample C	152,807	2,382,608	1,138,135			
30 min Sample A	7220	165,212	203,224	95.9	94.3	82.4
30 min Sample B	5364	97,893	115,183	97.3	96.7	91.8
30 min Sample C	9029	18,289	121,960	94.1	99.2	89.3
0 min (No NCCO) A	378,808	4,880,022	1,138,996			
0 min (No NCCO) B	328,253	4,116,698	1,135,041			
0 min (No NCCO) C	648,736	8,063,563	2,207,021			
30 min (No NCCO) A	353,599	4,144,521	1,249,259	6.7	15.1	− 9.7
30 min (No NCCO) B	356,349	4,716,458	1,123,240	− 8.6	− 14.6	1.0
30 min (No NCCO) C	553,028	6,415,579	1,525,011	14.8	20.4	30.9

A significant average reduction of 95.8%, 96.7% and 87.8% for Dronabinol, Cannabidiol and Cannabinol, respectively, can be observed after 30 minutes of NCCO treatment compared to very minimal reductions of 4.3%, 7.0%, and 7.4%, respectively, in control. This suggests that NCCO is highly efficient in eliminating air pollutants produced by marijuana smoke and the reduction is not caused by the natural absorption of these volatile compounds onto the exposed surfaces or decomposition.

### Indoor air quality parameters

**Fig. 2:**
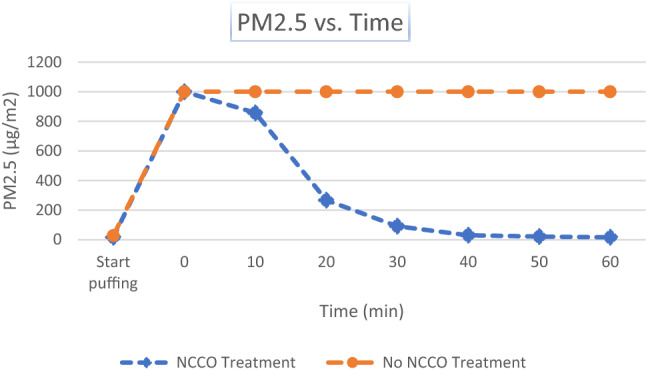
Particulate matter smaller than 2.5 microns from marijuana smoke

**Fig. 3:**
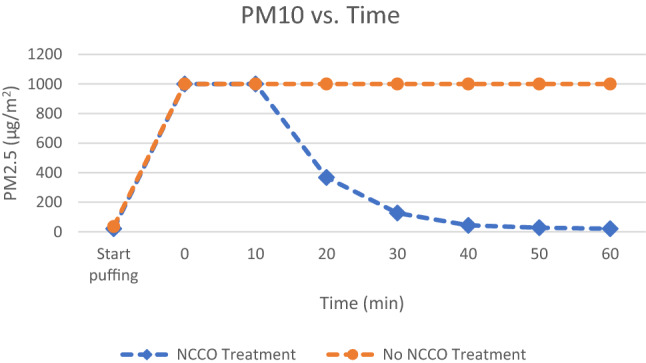
Particulate matter smaller than 10 microns from marijuana smoke

**Fig. 4:**
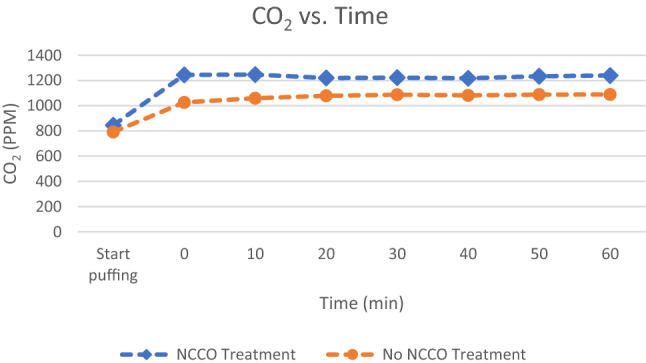
Carbon dioxide concentration from marijuana smoke

**Fig. 5:**
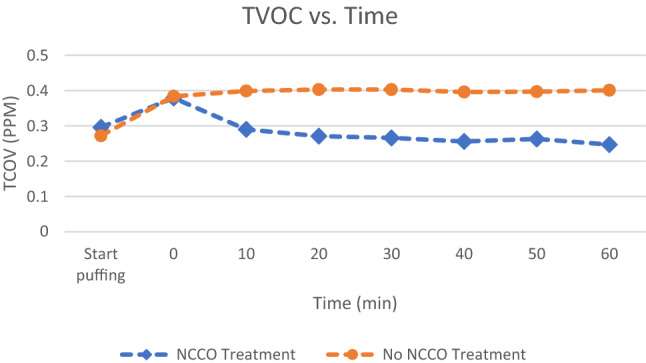
Total volatile organic compounds from marijuana smoke

Data collected by the Air Mentor 2 are tabulated in Appendix 1. Most air purifiers are equipped with HEPA filters which cannot efficiently remove fine particles and ultrafine particles. In this study, marijuana smoke generates high level of fine particulate matters in the air. The IAQ parameters measured by PM2.5 and PM10 for 2.5 microns and 10 microns particulate matters are shown in Figs. 2 and 3. Carbon Dioxide (CO_2_) and Total Volatile organic compounds (TVOC) measurements are shown in Figs. 4 and 5, respectively. All four graphs start with the baseline room IAQ measurements before the start of puffing a joint. Time zero refers to the end of puffing and beginning of the experiment. All concentrations should be at their maximum values. In the case of PM2.5 and PM10, the Air Mentor 2 can only measure up to 1000 µg/m^3^. This implies that both PM2.5 and PM10 in Figs. 2 and 3 have exceeded the maximum measurable by the device.

Cigarette smoke contains a high level of VOC’s, a primary source of dangerous chemicals such as benzene, styrene, toluene and xylenes. VOC’s have also been found to be released predominantly from marijuana leaves. [14]

### Relative odour intensity

**Fig. 6:**
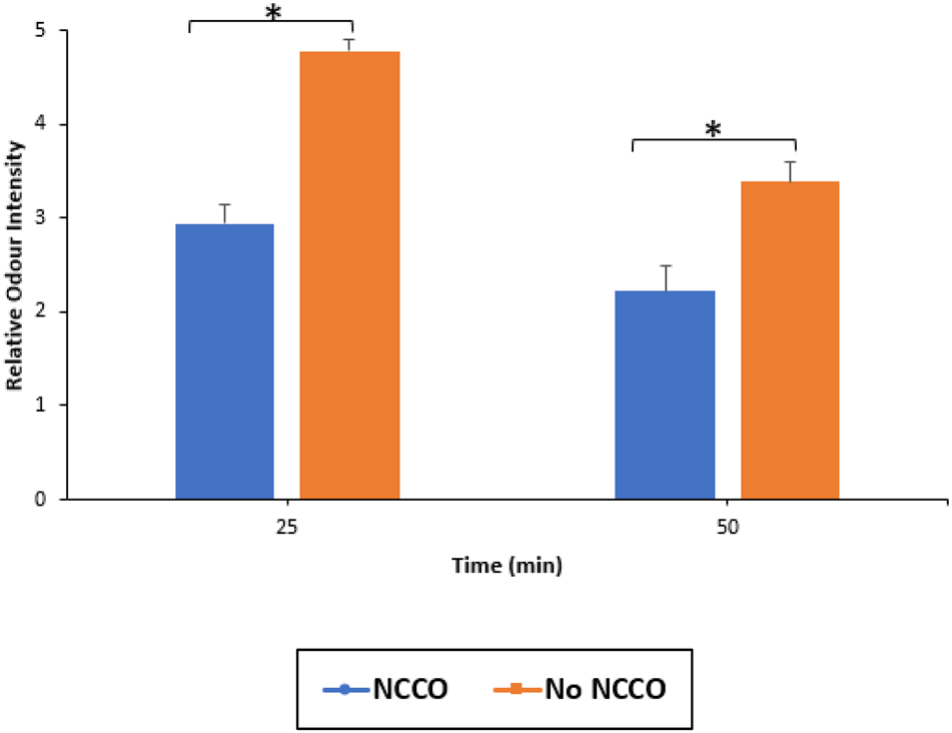
Summary of relative odour intensity (*n* = 18), error bar represents 1 SEM. *p < 0.05

**Fig. 7:**
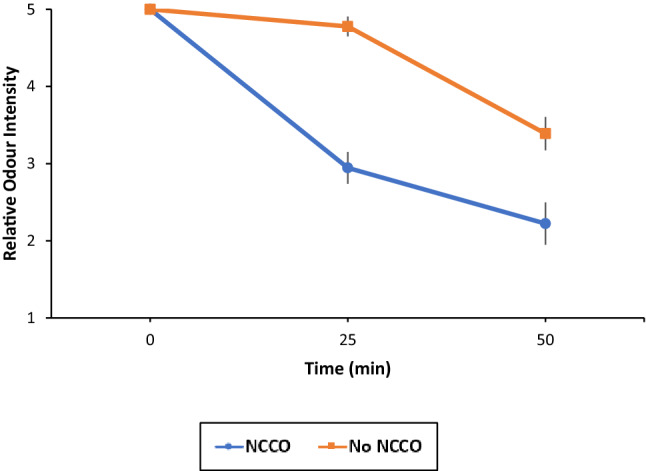
Summary of average relative odour intensity through treatment time. Error bar represents 1 SEM

Since all volunteers are non-cannabis users, they were exposed to the maximum odour of 5 and baseline odour of 1 in a first trial. The complete ratings submitted by each individual are available in Appendix 2. The relative average odour intensity perceived by volunteers after 25 and 50 minutes of NCCO treatment or control is summarized in Figs. 6 and 7.

The sample size of the odour assessment was reduced from 20 to 18 volunteers because two volunteers reported to have misunderstood the instructions. Both figures present the same data which demonstrate a significant reduction in relative marijuana odour after 25 and 50 minutes of NCCO treatment compared to the control group, indicating an effective reduction of odour intensity. Because of the standard error measurement, the drops in both samples are statistically significant.

## Discussion

The most objective measures for indoor air quality improvement after exposure to marijuana smoke are the actual concentration reduction in marijuana chemicals in the air. Table 2 shows the GC/MS measurements for the three representative compounds Dronabinol, Cannabidiol and Cannabinol. After 30 min of air purification using the NCCO air purifier, all three compounds are significantly reduced by up to 96.7% in the room. The controlled experiment basically shows a minimal reduction in all three compounds. The table shows both positive and negative percentage change in the compound concentrations. The negative percentage reduction implying an increase in the compound centration after 30 min is likely due to the nonuniform distribution of the compounds in the large experiment room. The overall average of the percentage reduction for the controlled experiment is only 6.2%. This suggests that the chemical compounds from marijuana smoke can stay in the air for long period of time, instead of quickly settling on or attached to surfaces. There is also no significant decomposition of these key marijuana chemicals. This further supports that marijuana odour can linger for a long period of time.

The NCCO technology clearly demonstrates its ability to destroy marijuana chemical compounds in the air by reducing the average chemical concentration to 93.4% for this setup. This percentage reduction is not universal as the air purification efficiency varies by the volumetric flowrate of the air purifier. However, Fig. 1 clearly shows that the percentage reduction is very impressive.

Another objective measure is the assessment of standard IAQ parameters. Appendix 1 contains all the raw data from the Air Mentor 2 detector. The room temperature was from 26 to 27 degrees Celsius. The relative humidity was from 54 to 56%. Both parameters did not vary much during the experiments. Figures 3 and 4 demonstrate that marijuana smoke generates a lot of fine particles in the air. These fine particles are more hazardous to human health. Their negative health impact is much worse when fine particles carry harmful chemicals, bacteria, or even viruses such as COVID-19. Wu et al. (2020) have concluded that “small increase in long-term exposure to PM2.5 leads to a large increase in the COVID-19 death rate.” [15] As stated earlier, the Air Mentor 2 detector can only measure up to 1000 µg/m^3^ for PM2.5 and PM10. This implies that both PM2.5 and PM10 in Figs. 2 and 3 have exceeded the maximum measurable by the device. Without the NCCO air purifier, PM2.5 and PM10 exceeded the maximum detectable value for the entire duration of the controlled experiment. While the efficiency of PM2.5 and PM10 reduction may be partly due to the HEPA filter in the air purifier, it is known that HEPA filters cannot completely capture all particulate matters. From both Figs. 2 and 3 and the raw data in Appendix 1, the NCCO air purifier reduced PM2.5 and PM10 back to the baseline pre-smoke level.

One of the by-products from the NCCO technology is carbon dioxide. Figure 4 shows that the CO_2_ level jumped at the beginning likely due to CO_2_ released from burning the joint. Afterwards, CO_2_ levels stay at similar level throughout the experiment and control. There is no noticeable increase in CO_2_ level released from the catalytic oxidation process.

VOC’s are known to be released from marijuana. Figure 5 demonstrates again that NCCO technology is able to reduce the TVOC level in the room.

There is no good objective measurement device for odour. Although electronic nose instruments are available, they need to be trained with qualified samples to build a database for reference. This poses a problem with measuring marijuana odour as many odours are made up of multiple different chemicals and/or molecules. The large variety of marijuana species and supplies make standardized odour measurement even more challenging. This is the reason why a sample population is used to assess odour reduction in this study. Figures 6 and 7 show statistically significant reduction in odour at 25 min and further statistically significant reduction at 50 min. Both the experiment and control show reductions. The relative odour intensity of marijuana did not experience a reduction as significant as observed in its chemical concentration. This may be attributed to the volunteer group consisting of non-regular cannabis users. Some volunteers commented that the odour of marijuana is so unpleasant that any presence of odour is strong or intense, thus, there may be a bias towards rating at a higher score due to difficulties in distinguishing strong and weak odour. Nevertheless, the NCCO technology has been shown to reduce the odour level from the odour intensity of 5 to 2.22, a drop of 55.6% after 50 min of air purification.

## Conclusions

Cannabis smoke produces a complex profile of air pollutants. The NCCO technology has demonstrated effective reductions in concentrations of key marijuana compounds and indoor air quality standard parameters. The average reduction in Dronabinol, a pharmaceutical form of THC, CBD and Cannabinol, reaches 93.4%, while a lack of air purification reduces compound levels by 6.2%. The technology also demonstrates statistically significant reduction in PM2.5, PM10 and TVOC generated from marijuana smoke. Furthermore, the in-person odour assessment also confirmed statistically significant reductions in marijuana odour by 55.6% after 50 min of air purification.

## Appendix 1: Air quality parameters during sample collection for chemical analysis

NCCO treatment.

**Table:**

Time (min)	PM2.5 (µg/m^2^)	PM10 (µg/m^2^)	CO_2_ (PPM)	TCOV (PPM)	Temperature (℃)	Humidity %
Room air	16	21	845	0.296	27	54
0	1000	1000	1245	0.379	27	55
10	857	1000	1247	0.290	27	56
20	267	368	1220	0.271	27	56
30	91	127	1223	0.266	26	56
40	30	44	1218	0.256	26	56
50	21	28	1234	0.263	26	56
60	15	20	1240	0.247	26	56

No NCCO treatment.

**Table:**

Time (min)	PM2.5 (µg/m^2^)	PM10 (µg/m^2^)	CO_2_ (PPM)	TCOV (PPM)	Temperature (℃)	Humidity %
Baseline	27	36	791	0.272	27	53
0	1000	1000	1026	0.384	27	54
10	1000	1000	1059	0.399	27	55
20	1000	1000	1079	0.403	27	55
30	1000	1000	1089	0.403	27	54
40	1000	1000	1081	0.396	27	54
50	1000	1000	1088	0.397	27	54
60	1000	1000	1089	0.401	27	54

## Appendix 2: Data table of relative odour intensity

**Table:**

Treatment	NCCO		No NCCO	
Time (min)	25	50	25	50
Volunteer 1	2	1	3	2
Volunteer 2	3	1	5	2
Volunteer 3	2	1	5	4
Volunteer 4	3	3	5	4
Volunteer 5	2	2	5	3
Volunteer 6	3	4	5	4
Volunteer 7	2	2	5	2
Volunteer 8	4	4	5	3
Volunteer 9	2	2	5	3
Volunteer 10	3	2	4	3
Volunteer 11	3	1	5	2
Volunteer 12	3	1	5	4
Volunteer 13	3	3	4	5
Volunteer 14	4	4	5	4
Volunteer 15	4	4	5	4
Volunteer 16	2	1	5	4
Volunteer 17	3	2	5	4
Volunteer 18	5	2	5	4
